# Centimeter-Deep NIR-II Fluorescence Imaging with Nontoxic AIE Probes in Nonhuman Primates

**DOI:** 10.34133/2020/4074593

**Published:** 2020-09-26

**Authors:** Zonghai Sheng, Yaxi Li, Dehong Hu, Tianliang Min, Duyang Gao, Jen-Shyang Ni, Pengfei Zhang, Yuenan Wang, Xin Liu, Kai Li, Hairong Zheng, Ben Zhong Tang

**Affiliations:** ^1^Paul C. Lauterbur Research Center for Biomedical Imaging, Key Laboratory for Magnetic Resonance and Multimodality Imaging of Guangdong Province, Shenzhen Key Laboratory of Ultrasound Imaging and Therapy, CAS Key Laboratory of Health Informatics, Institute of Biomedical and Health Engineering, Shenzhen Institute of Advanced Technology, Chinese Academy of Sciences, Shenzhen 518055, China; ^2^Department of Biomedical Engineering, Southern University of Science and Technology (SUSTech), Shenzhen 518055, China; ^3^Department of Radiation Oncology, National Cancer Center, National Clinical Research Center for Cancer, Cancer Hospital & Shenzhen Hospital, Chinese Academy of Medical Sciences and Peking Union Medical College, Shenzhen 518055, China; ^4^Department of Chemistry, The Hong Kong University of Science & Technology, Clear Water Bay, Kowloon, Hong Kong, China

## Abstract

Fluorescence probes with aggregation-induced emission (AIE) characteristics are of great importance in biomedical imaging with superior spatial and temporal resolution. However, the lack of toxicity studies and deep tissue imaging in nonhuman primates hinders their clinical translation. Here, we report the blood chemistry and histological analysis in nonhuman primates treated with AIE probes over tenfold of an intravenous dose of clinically used indocyanine green (ICG) during a study period of 36 days to demonstrate AIE probes are nontoxic. Furthermore, through bright and nontoxic AIE probes and fluorescence imaging in the second window (NIR-II, 1,000–1,700 nm), we achieve an unprecedented 1.5-centimeter-deep vascular imaging in nonhuman primates, breaking the current limitation of millimeter-deep NIR-II fluorescence imaging. Our important findings, i.e., nontoxic features of AIE probes and centimeter-deep NIR-II vascular imaging in nonhuman primates, may facilitate successful translation of AIE probes in clinical trials.

## 1. Introduction

Fluorescence imaging with organic molecules as probes has become an essential tool for bench researchers and clinicians interested in understanding disease mechanism or improving human health due to excellent spatial and temporal resolution [1–3]. However, the imaging sensitivity and spatial resolution can be generally weakened due to the aggregation-caused quenching (ACQ) of fluorescence probes at high concentrations or condensed states [4]. Fortunately, twenty years ago, our team discovered a specific class of organic molecules emitting bright fluorescence even in the aggregated state; this phenomenon of fluorescence emission in the aggregated state is termed as aggregation-induced emission (AIE) [5]. Since then, numerous AIE molecules have been synthesized as fluorescence probes for biomedical imaging research [6–10]. AIE probes exhibit excellent biocompatibility, high quantum yield (QY), tunable emission wavelength, large stoke shift, and great optical stability, enabling their use in next-generation fluorescence materials for biomedical imaging [11, 12]. Recently, our team and others utilized the bandgap engineering strategy to further extend the emission wavelength of AIE probes from the first near-infrared (650–950 nm, NIR-I) to the second near-infrared (1,000–1,700 nm, NIR-II) window and achieve deeper tissue imaging [13–18]. NIR-II fluorescence imaging with existing probes including some AIE probes has already demonstrated the ability of millimeter-deep tissue imaging in the mouse models through suppressed scatters of photons in tissue and diminished autofluorescence background [19–24]. However, challenges remain in realizing the clinical translation of AIE probes and imaging depths greater than the current limitation of millimeters in the NIR-II window.

Recently, Carr et al. reported NIR-II fluorescence imaging with the clinically approved contrast agent indocyanine green (ICG) [25]. This was followed by the first clinical trial which was performed for NIR-II fluorescence imaging-guided liver tumor surgery with ICG as the contrast agent based on its reliable safety and extensive practice [26]. The successful clinical implementation of ICG inspired us to focus on toxicity studies of AIE probes and the development of unprecedented centimeter-deep *in vivo* imaging application in the NIR-II window using these probes. Nonhuman primate studies are known to facilitate clinical trials because of the evolutionary relationship between nonhuman primates and humans [27, 28]. Previous studies in nonhuman primates have reported toxicity evaluation of fluorescence probes, including quantum dots [29], silicon nanoparticles [30], gold nanoparticles [31], and nanodiamonds [32]. These studies have enhanced our understanding on the toxicity of fluorescence probes, providing significant implications for the studies of novel AIE probes [33–35]. Therefore, we speculated that preclinical studies in a nonhuman primate could facilitate in-depth understanding on the toxicity of synthetic AIE probes. Furthermore, preclinical trials involving nonhuman primates involve lower risk and reduced cost compared to human clinical trials and must be performed before trials in human. Moreover, we sought to increase NIR-II fluorescence imaging depth from millimeter to centimeter level. The bright AIE characteristics and NIR-II imaging window could facilitate deeper tissue imaging in nonhuman primates.

Here, we report the toxicity profile of AIE probes with high QY in three cynomolgus monkeys, one of the important nonhuman primates, over a study period of 36 days following the intravenous (IV) injection of 16 mg kg^−1^ AIE probes. One of them was subsequently euthanized for *ex vivo* histological analysis, and the other two were observed for further ten months. *In vivo* hematological and tissue compatibility analysis revealed that AIE probes are highly biocompatible. These bright and nontoxic AIE probes were successfully applied in NIR-II fluorescence imaging of the blood vessels and lymph nodes, achieving unprecedented 1.5-centimeter depth and high resolution in nonhuman primates. Our pilot study demonstrated ample clinical potential of nontoxic AIE probes in noninvasive biomedical imaging.

## 2. Results and Discussion

### 2.1. Synthesis and Characterization of AIE Probes

**Figure 1:**
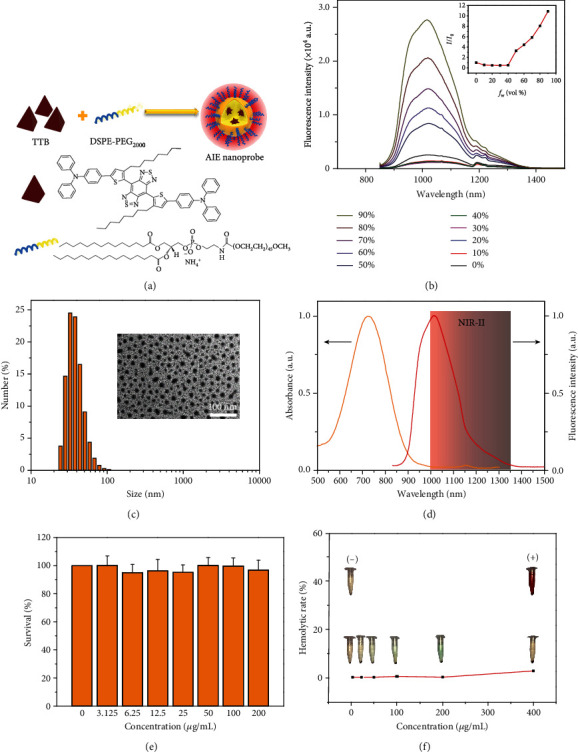
Characterization and *in vitro* biocompatibility evaluation of AIE probes. (a) Schematic illustrations of preparation of AIE probes through a nanoprecipitation method. (b) Fluorescence spectra of TTB molecules in THF solution with different water fractions (vol%). The inset shows plots of the AIE curves of TTB. (c) Dynamic light scattering data of the aqueous AIE probes. Inset: transmission electron microscopy image of water-dispersed AIE probes. (d) Fluorescence and absorption spectra of AIE probes. The red-shaded area shows the NIR-II emission wavelength of AIE probes from 1000 to 1350 nm and the maximum emission wavelength at 1050 nm. (e) The cytotoxicity analysis of the AIE probes by routine MTT assay. (f) Hemolysis analysis of red blood cells after exposure to AIE probes at 0 *μ*g mL^−1^, 25 *μ*g mL^−1^, 50 *μ*g mL^−1^, 100 *μ*g mL^−1^, 200 *μ*g mL^−1^, and 400 *μ*g mL^−1^. The inset shows digital photographs of AIE probe-treated red blood cells. The negative control group (-) and positive control group (+) are phosphoric acid buffer-treated red blood cells and deionized water-treated red blood cells, respectively.

A typical NIR-II AIE molecule shown in Figure 1(a), 4,7-di(4-(4-octylthiophen-2-yl)-N,N-diphenylaniline)-benzo[1,2-c:4,5-c′]bis([1,2,5]thiadiazole) (TTB) with a donor-acceptor-donor structure, was designed and synthesized by a two-step chemical reaction, and the products were subsequently characterized (Fig. S1–S7). The AIE feature of the as-prepared TTB molecule was evaluated by adding different amounts of tetrahydrofuran in water (Figure 1(b)). The results suggested that the fluorescence intensity of TTB gradually decreased with the rising water volume fraction (*f*_w_) from 0 to 40 vol% and then increased when *f*_w_ was further raised from 40 to 90 vol%, confirming the AIE signature [36]. The TTB was demonstrated to be a NIR-II fluorophore due to the maximum fluorescence emission at 1050 nm (Figure 1(b)). To endow TTB with water solubility and enhanced brightness, DSPE-PEG_2000_, an amphiphilic polymer matrix and a type of clinically used excipient, was adopted to encapsulate numerous TTB molecules to form AIE probes (Figure 1(a)). The whole process is facile and efficient for large-scale fabrication without sophisticated equipment, supporting its potential for approval in clinical studies and applications. The produced AIE probes exhibited a spherical morphology with an average diameter of 35 nm and excellent monodispersity (Figure 1(c)). Moreover, AIE probes readily dispersed in the buffer solution without aggregations for more than six months (Fig. S8). They had a maximum absorption peak at 728 nm, an emission peak at 1050 nm, and the emission tail at 1350 nm, indicating representative NIR-II probes (Figure 1(d)). The fluorescence QY was ~10%, which was higher than most of the NIR-II fluorophores reported previously using IR-26 dye as a reference (Table S1 and Fig. S9). The excellent photostability of AIE probes in PBS was observed under continuous exposure of natural light for one month (Fig. S10). Furthermore, Cell Counting Kit (CCK-8) assay was applied for evaluating the cytotoxicity of AIE probes in endothelial cells of the human umbilical vein (Figure 1(e)). The assessment revealed low cytotoxicity of the AIE probes, as the cell viability remained above 95% after incubation with 200 *μ*g mL^−1^ of AIE probes for 24 hours, suggesting low cytotoxicity. We further assessed the blood compatibility of AIE probes in cynomolgus monkeys using hemolytic analysis (Figure 1(f)). The hemolytic rate of blood samples incubated with AIE probes of 0 *μ*g mL^−1^, 25 *μ*g mL^−1^, 50 *μ*g mL^−1^, 100 *μ*g mL^−1^, 200 *μ*g mL^−1^, and 400 *μ*g mL^−1^ AIE probes for 24 hours was below 5%, indicating its excellent *in vitro* biocompatibility. These results suggested that the uniform, bright, stable, and *in vitro* biocompatible AIE probes designed in the present study could be an excellent candidate for NIR-II fluorescence imaging.

### 2.2. *In Vivo* Toxicity Evaluation in Cynomolgus Monkeys

**Figure 2:**
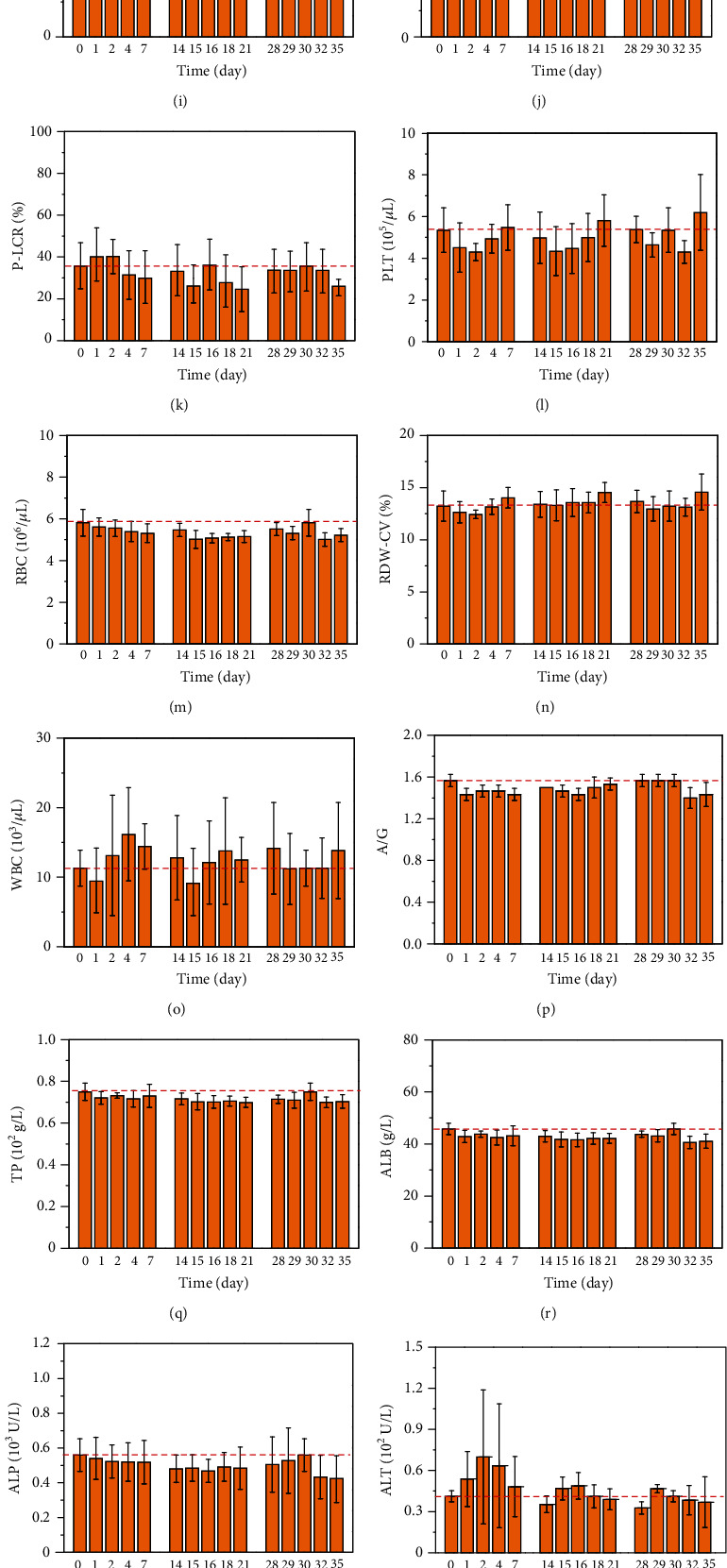
Hematological test and serum biochemistry results for treated cynomolgus monkeys. (a) Schematic illustrations of acute toxicity evaluation of the AIE probes with three different IV doses in three healthy adult cynomolgus monkeys over 36 days. (b–z-1) The results (*n* = 3) show no effect on the immune system, inflammatory response, and liver and kidney functions. The red dashed line indicates the normal baseline of cynomolgus monkeys. Error bars represent one standard deviation above the mean. Abbreviations and reference range: hematocrit: HCT (35.4-54.4%); hemoglobin: HGB (11.3-16.7 g dL^−1^); lymphocyte: LYM (9.8-86.9%), LYM (1.0‐11.7 × 10^3^ *μ*L^−1^); mean corpuscular hemoglobin: MCH (15.8-24.9 pg); mean corpuscular hemoglobin concentration: MCHC (27.2-33.5 g dL^−1^); mean corpuscular volume: MCV (57.5-82.2 fL); mean platelet volume: MPV (8.3-13.7 fL); plate volume distribution width: PDW (9.3-21.9 fL); platelet large cell ratio: P-LCR (10.1-56.8%); platelet: PLT (275‐742 × 10^3^ *μ*L^−1^); red blood cell: RBC (4.3‐7.8 × 10^6^ *μ*L^−1^); red cell distribution width-CV: RDW-CV (11-14.7%); white blood cell: WBC (1.7‐30.3 × 10^3^ *μ*L^−1^); albumin globulin ratio: A/G (0.84-1.57); total protein: TP (72-98.3 g L^−1^); albumin: ALB (41.1-50.5 g L^−1^); alkaline phosphatase: ALP (261.2-2265.0 U L^−1^); alanine aminotransferase: ALT (2.2-283.7 U L^−1^); aspartate aminotransferase: AST (22.7-243.4 U L^−1^); direct bilirubin: DBIL (0.3-1.2 *μ*mol L^−1^); gamma glutamyl transferase: GGT (35.64-106.73 U L^−1^); globulin: GLOB (29.3-46.3 g L^−1^); total bilirubin: TBIL (1.14-7.1 *μ*mol L^−1^); creatinine: CREA (62-120 *μ*mol L^−1^); urea: UREA (2.9-6.1 mmol L^−1^).

**Figure 3:**
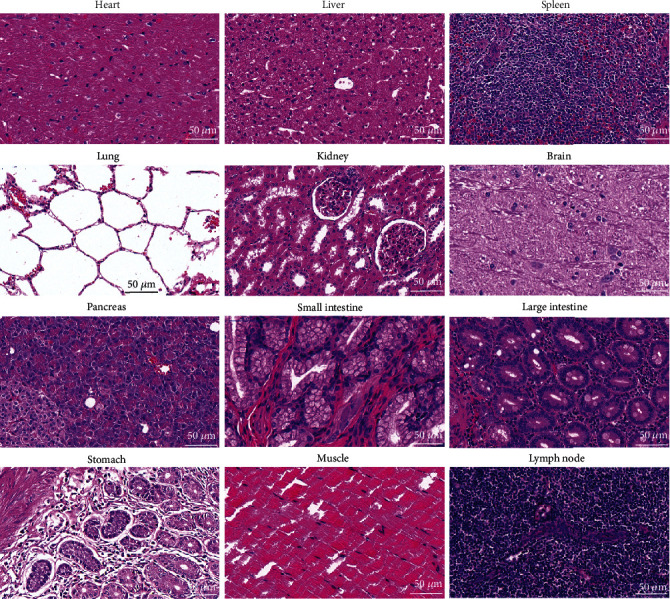
Histological images of the major organs of one randomly selected cynomolgus monkey at the end of 36 days after IV injection of AIE probes. Evaluations were performed by two pathologists, and no anomalies were observed. Tissues were collected from the heart, liver, spleen, lung, kidney, brain, pancreas, small intestine, large intestine, stomach, muscle, and lymph node. Images were taken at ×40 magnification with standard hematoxylin and eosin staining.

Before performing *in vivo* imaging, we designed a dose escalation experiment using three dose schemes (1, 5, and 10 mg kg^−1^) over 35 days to further evaluate *in vivo* biocompatibility in three healthy adult cynomolgus monkeys (Figure 2(a)). No IV injection of AIE probes in cynomolgus monkeys has been reported to our knowledge. Therefore, the escalating of IV injection dose of AIE probes could effectively investigate the safety of the monkeys and detect the tolerated ability of AIE probes. The cumulative IV dose reaches 16 mg kg^−1^ (equivalent IV dose of an adult human ~5.3 mg kg^−1^, 1 : 3 = human IV dose : monkey IV dose), which was more than tenfold of the IV dose of ICG (0.5 mg kg^−1^) for NIR-II fluorescence imaging in clinical trials [25, 26]. Therefore, the designed dose-escalating experiment could be used to evaluate the tolerance of as-prepared AIE probes in cynomolgus monkeys. Before IV injection of AIE probes, physiological parameters and blood chemistry of three cynomolgus monkeys were measured as the control group. This experiment design can not only greatly minimize the error caused by individual differences but also potentially reduce the sample size of experimental monkeys. After IV injection of AIE probes with the dosage escalation of 1, 5 mg kg^−1^, and 10 mg kg^−1^, the body weight of the monkeys showed no fluctuation, indicating minimal systemic effects (Fig. S11). Similarly, there was no change in body temperatures during the test (Fig. S12). No statistically significant differences in daily behavioral patterns (eating, drinking, sleeping, and activity), neurological status, and physical features were observed (data not shown). Next, a complete blood count was conducted at regular intervals to investigate the immune response to AIE probes (Figures 2(d)–2(q)). Results showed no sharp fluctuation in the 14 hematological indicators compared with the control and reference values, indicating no acute toxicity. Similarly, serum biochemistry assays revealed that 10 indicators of liver function (TP, A/G, ALB, ALP, ALT, AST, DBIL, GGT, GLOB, and TBIL) and 2 indicators of kidney function (CREA and UREA) were normal throughout the examination period (Figures 2(p)–2(z-1)), suggesting no sign of liver and kidney injuries. At the end of the 36-day postinjection period, one cynomolgus monkey was randomly selected and euthanized, and its heart, liver, spleen, lung, kidney, brain, muscle, and lymph nodes were removed for histological analysis (Figure 3). Two clinical pathologists, both unaware of the AIE probe treatment, analyzed the tissue sections and found no abnormality in the myocardial fibers, no signs of inflammatory response in the liver, no pulmonary fibrosis in the lung, nor necrosis in all histological samples, and clear structure of the glomerular. These results provided preliminary evidence that AIE probes, with a cumulative IV dose of 16 mg kg^−1^, did not induce acute toxicity in cynomolgus monkeys during the experimental period. Moreover, two other AIE probe-treated cynomolgus monkeys have been raised for more than ten months without any adverse response.

### 2.3. *In Vivo* Biodistribution and Metabolism of AIE Probes

During the experiments, we detected fluorescence signals in fecal samples at different time points (Fig. S13), demonstrating that AIE probes could be gradually excreted from the body through feces. The clearance mechanism of AIE probes *in vivo* was further investigated to analyze their biodistribution and metabolism by *ex vivo* fluorescence imaging. The results showed that AIE probes were predominantly accumulated in the liver, spleen, and lymph nodes (Fig. S14). Conversely, there was no fluorescence signal observed in the muscle, stomach, brain, heart, intestine, kidney, and lung (Fig. S15), which suggested that AIE probes, like other nanoparticles with similar size and surface properties, were primarily recognized by the reticuloendothelial system [28]. Although most AIE probes were accumulated in the liver, hepatic function remained normal during our experimental period, further demonstrating no evidence of acute toxicity. We will optimize the design of AIE probes and polymer matrix to improve their *in vivo* clearance efficiency in the future.

### 2.4. *In Vivo* NIR-II Fluorescence Perfusion Imaging

**Figure 4:**
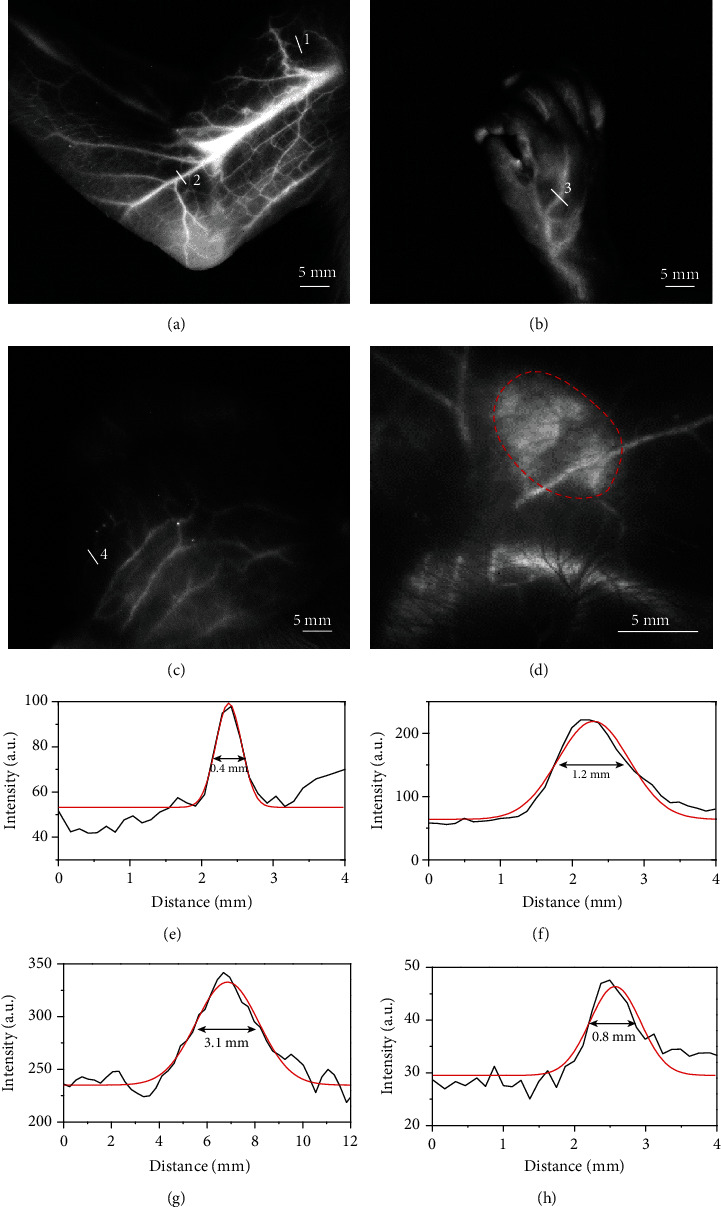
NIR-II fluorescence imaging of the blood vessels and lymph nodes in the cynomolgus monkey after IV injection of the AIE probes. Before performing NIR-II fluorescence imaging, we removed all hair from the monkey's inner arm and scalp but kept hair from the hand. NIR-II fluorescence imaging of the arm (a), the hand (b), and scalp vasculature (c) in the cynomolgus monkey. (d) NIR-II fluorescence imaging of an axillary lymph node marked by the red dotted line. The fluorescence imaging parameters: 808 nm laser with a power density of 30 mW cm^−2^, 1250 nm long-pass filter, 200 ms exposure time using a 640 × 512 pixel two-dimensional InGaAs/SWIR camera (Photonic Science, UK), and IV dose of 2 mg kg^−1^. (e–h) Cross-sectional NIR-II fluorescence intensity profiles of vasculature marked in white lines 1, 2, 3, and 4.

To exploit perfusion imaging of AIE probes, we employed a commercial NIR-II imaging system with an ordinary InGaAs camera, an 808 nm excitation laser (30 mW cm^−2^), and a 1250 nm long-pass filter to acquire vasculature information in a cynomolgus monkey. Shortly after the IV injection of AIE probes (dose: 2 mg kg^−1^), we collected video-rate fluorescence images to record vascular perfusion at 5 frames per second (Supplementary Video 1, Fig. S16). Even though our imaging speed of the camera was much slower than that of previously reported liquid nitrogen-cooled InGaAs camera [37], yet, we still achieved clear images of vascular perfusion due to the contrast enhancement of bright AIE probes in the cynomolgus monkey. Within the 138.5 s postinjection period, the AIE probes had fully perfused into the arteries and veins of the forearm and markedly outlined the superficial vasculature (Figure 4(a)). Moreover, this vascular perfusion imaging can also be performed in the absence of depilation (Figure 4(b) and Fig. S17), which was evident of excellent imaging quality with suppressed scatter and absorption of a photon and diminished autofluorescence background in the NIR-II window [38]. This perfusion imaging can also be applied to visualize the capillaries in the scalp of monkeys noninvasively (Figure 4(c)). The diameter of the arteries at representative locations ranged from 0.4 mm to 3.1 mm based on the Gaussian-fitting full width at half maximum (FWHM) of the cross-sectional profiles (Figures 4(e)–4(h)). In addition to NIR-II fluorescence angiography, we visualized the axillary lymph nodes of the cynomolgus monkey using AIE probes after 5 min of subcutaneous injection (0.5 mL and 1 mg mL^−1^). The size of the axillary lymph nodes was ~0.7 cm × 0.5 cm (dashed red circle in Figure 4(d)), which was consistent with the *in vitro* data (Fig. S14). This result demonstrated great clinical potential of AIE probes in NIR-II fluorescence imaging-guided sentinel lymph node biopsy for cancer diagnosis and treatment [39, 40].

### 2.5. In Vivo Centimeter-Deep Fluorescence Vascular Imaging

**Figure 5:**
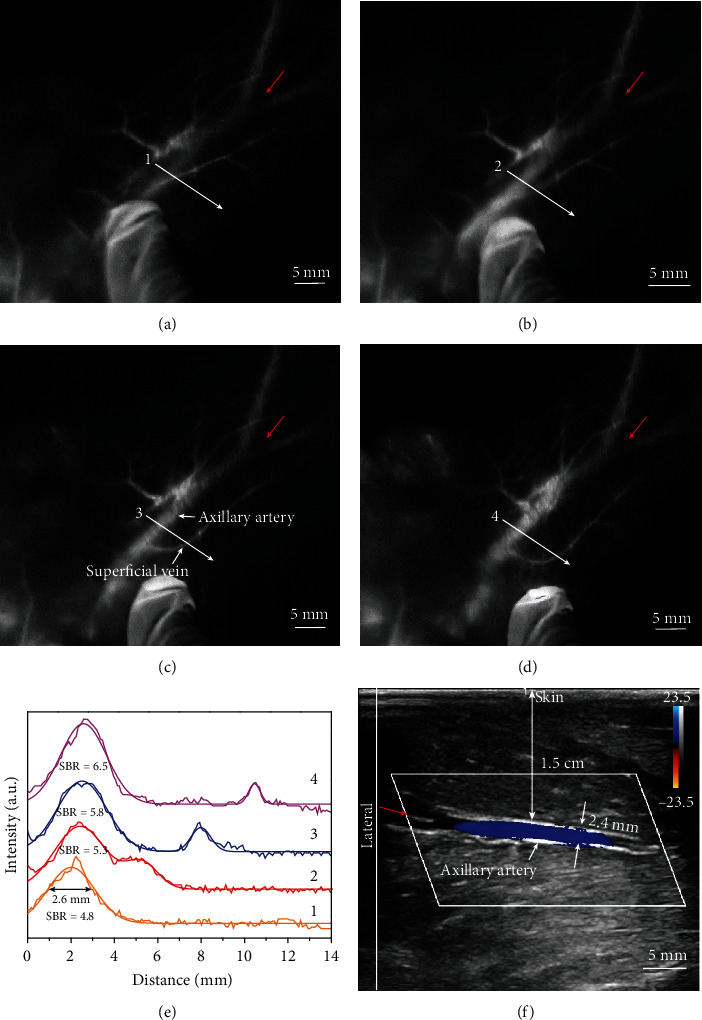
NIR-II fluorescence imaging of the deep axillary artery in a healthy adult cynomolgus monkey after IV injection of AIE probes. (a–d) Changes in the relative position of the superficial vein on the skin and the deep axillary artery. When we moved the epidermis, the superficial vein was moved accordingly; however, the deep axillary artery remained stationary. The fluorescence imaging parameters: 808 nm laser with a power density of 30 mW cm^−2^, 1250 nm long-pass filter, 200 ms exposure time using a 640 × 512 pixel two-dimensional InGaAs/SWIR camera, and intravenous dose of 2 mg kg^−1^. (e) Cross-sectional profiles of NIR-II fluorescence imaging of the same axillary artery shown in (a–d) (red arrows indicates the same bifurcation in the axillary artery). (f) Ultrasonic color Doppler imaging of the axillary artery of the cynomolgus monkey, indicating the depth of the same axillary artery at ~1.5 cm (red arrow indicates the same bifurcation in the axillary artery as shown in Figures 4(a)–4(d)).

Vascular imaging in deep tissue is critical for the diagnosis of cardiovascular diseases and tumors [41]. Following the successful demonstration of AIE probes in superficial tissue with NIR-II imaging, we conducted a more challenging centimeter-penetration vascular imaging in the cynomolgus monkey (Figures 5(a)–5(d)). Here, we purposedly selected the axillary artery of the cynomolgus monkey to perform the NIR-II fluorescence imaging. Prior to AIE probe injection, the depth and size of the axillary artery were measured using ultrasonic color Doppler imaging, confirming the artery located ~1.5 cm beneath the skin with a diameter of 2.4 mm (Figure 5(f)). We chose the bifurcation of the axillary artery as the landmark (shown as the red arrow in Figure 5(c)) for both ultrasound and fluorescence imaging. After the IV injection of AIE probes (2 mg kg^−1^), we recorded the video-rate NIR-II fluorescence vascular imaging, illustrating the stationary 1.5 cm-deep axillary artery and the moveable superficial veins while pushing the skin (Supplementary Video 2). By measuring the Gaussian-fitting FWHM of the cross-sectional profiles, we observed no change in the position of the deep arteries while the superficial veins gradually moved away from the skin pushing direction (Figure 5(e)). The imaging signal-to-background ratio of the deep arteries increased from 4.8 to 6.5 due to skin lifting effect. The apparent diameter of the artery was 2.6 ± 0.2 mm (Figure 5(e)), which was consistent with the ultrasound measurement. This indicated that we achieved noninvasive vascular imaging at 1.5 cm depth in nonhuman primates using the AIE probes for the first time.

## 3. Conclusions

In conclusion, cynomolgus monkeys were IV injected with a cumulative dose of 16 mg kg^−1^ of designed AIE probes and survived without any adverse response. All measured physiological parameters and hematological markers in treated cynomolgus monkeys were in the normal range, and the histological analysis of the major organs did not reveal any acute toxicity. The AIE probes trapped in the liver, spleen, and lymphatic system could be excreted through feces. The findings indicated that the organic AIE probes can be considered safe for nonhuman primates on the timescale of our experiment. Furthermore, through NIR-II fluorescence imaging with bright AIE probes, we achieved the unprecedented 1.5 cm-deep vascular imaging with high resolution and high contrast. These two distinguished features of NIR-II AIE probes, nontoxic and centimeter-deep fluorescence imaging, enable them to become a promising fluorophore candidate for fluorophores for angiography and lymphadenopathy. Therefore, our preliminary findings in this study could pave the way to promote clinical translation of NIR-II AIE probes in human clinical trials in the near future.

## 4. Materials and Methods

### 4.1. Materials and Characterization

All reagents were obtained commercially and used without further purification, except tetrahydrofuran (THF), dichloromethane (DCM), and toluene, which were purified before use. All air- and moisture-sensitive reactions were carried out in flame-dried glassware under argon protection. ^1^H (400 MHz) and ^13^C (100 MHz) NMR spectra were recorded on Bruker AV400 spectrometers (Bruker, Billerica, MA, USA). ^1^H NMR and ^13^C NMR spectra used tetramethylsilane (TMS) as an internal standard in CDCl_3_. High-resolution mass spectra were measured using Q-Exactive with Dionex Ultimate 3000 (Thermo Fisher Scientific, Waltham, MA, USA). UV-Vis-NIR absorption spectra were collected with a Shimadzu UV-2600 spectrophotometer. Fluorescence spectra were measured with a fluorescence spectrometer (F900, Edinburgh Instruments Ltd.). The NIR-II fluorescence imaging experiments were conducted by using NIR-OPTICS Series III 900/1700 small animal imaging system (Suzhou NIR-OPTICS Co., Ltd., China). Ultrasonic color Doppler imaging was performed using the Resona 7 ultrasound system with a L11-3U ultrasonic probe (Mindray Medical International Co., Ltd., China).

### 4.2. Synthesis of 4-(4-Octylthiophen-2-yl)-N,N-diphenylaniline 2

A solution of 3-octylthiophene 1 (1.96 g, 10.0 mmol) in THF (40 mL) was dropwise added with 2.5 Mn-BuLi (4.8 mL, 12.0 mmol) at -78°C. The mixture was stirred for 3 h under nitrogen protection. Then, SnBu_3_Cl (6.51 g, 20 mmol) was added to the reaction mixture and stirred overnight at room temperature. The mixture was quenched with water and washed with ethyl acetate. The combined organic layer was dried over anhydrous Na_2_SO_4_ and concentrated. The residual was dissolved in toluene as 1 M solution. The 1 M solution (9.6 mL) was added with 4-bromo-N,N-diphenylaniline (1.56 g, 8 mmol) and Pd (PPh_3_)_4_ (0.092 g, 0.08 mmol) and dissolved in toluene (80 mL) under nitrogen protection. After refluxing for 24 h and then cooling to room temperature, the mixture was quenched with water and washed with ethyl acetate. The combined organic phase was dried over anhydrous Na_2_SO_4_ and evaporated in vacuo. The crude product was subjected to column chromatography on silica gel with PE/EA 20 : 1 to 2 as a light yellow oil (2.36 g, 67%). ^1^H NMR (400 MHz, CDCl_3_) *δ* 7.45-7.43 (m, 2H), 7.27-7.25 (m, 4H), 7.12-7.01 (m, 9H), 6.79 (d, *J* = 0.8 Hz, 1H), 2.59 (t, *J* = 7.2 Hz, 2H), 1.71-1.66 (m, 2H), 1.43-1.32 (m, 10H), 0.93 (t, *J* = 7.2 Hz, 3H). ^13^C NMR (100 MHz, CDCl3) *δ* 147.60, 147.09, 144.26, 143.80, 129.30, 129.21, 128.99, 126.53, 124.41, 124.19, 123.89, 123.73, 122.98, 118.72, 31.92, 30.68, 30.48, 29.47, 29.37, 29.30, 27.88, 26.87, 22.70, 17.55, 14.13, 13.62. HRMS (ESI): *m*/*z* [M+H+] calculated for C_30_H_34_NS 440.2406; found: 440.24050.

### 4.3. Synthesis of TTB

A solution of 2 (2.20 g, 5.0 mmol) in THF (20 mL) was dropwise added with Mn-BuLi (2.4 mL, 6.0 mmol) at -78°C. The mixture was stirred for 3 h under nitrogen protection. Then, the reaction mixture was added with SnBu_3_Cl (3.26 g, 10 mmol) and stirred overnight at room temperature. The mixture was quenched with water and washed with ethyl acetate. The combined organic layer was dried over anhydrous Na_2_SO_4_ and concentrated. The residual was solved in toluene as 1 M solution. The 1 M solution (2.1 mL, 2.3 equiv.) was added with 4,7-dibromobenzo[1,2-c:4,5-c′]bis([1,2,5]thiadiazole) BBTD (0.307 g, 0.9 mmol) and beta-4-platinum (0.100 g, 0.09 mmol, 0.1 equiv.) and dissolved in toluene (5 mL) under nitrogen protection. After refluxing for 24 h and then cooling to room temperature, the mixture was quenched with 1 M KF aqueous solution and washed with ethyl acetate. The combined organic phase was dried over anhydrous Na_2_SO_4_ and evaporated. The crude product was subjected to column chromatography on silica gel with PE/EA 10 : 1 to TTB as a black green solid (350 mg, 47%). ^1^H NMR (400 MHz, CDCl3) *δ* 7.57 (d, *J* = 8.4 Hz, 4H), 7.36 (s, 2H), 7.30-7.26 (m, 9H), 7.15-7.03 (m, 15H), 2.58 (t, *J* = 7.6 Hz, 4H), 1.63-1.61 (m, 8H), 1.34-1.26 (m, 8H), 1.18-1.10 (m, 8H), 0.81 (t, *J* = 7.2 Hz, 6H). ^13^C NMR (100 MHz, CDCl3) *δ* 153.24, 147.60, 147.45, 147.00, 145.68, 129.36, 128.18, 126.78, 124.80, 124.67, 123.39, 123.22, 116.08, 31.79, 30.51, 30.35, 29.73, 29.46, 29.31, 29.16, 22.64, 14.12. HRMS (ESI): *m*/*z* [M+] calculated for C_66_H_64_N_6_S_4_ 1068.40686; found: 1068.40698.

### 4.4. NIR-II QY Test

The NIR-II fluorescence QY of the TTB-based AIE probes was measured according to the previous reports [24]. Generally, the absorption and emission spectra of AIE probes and IR-26 dye with five different concentrations at below OD 0.1 at 808 nm were measured, respectively. The integrated fluorescence (808 nm excitation) was plotted against absorbance for both AIE probes and IR-26 dye. The comparison of the slopes led to the measurement of the QD of AIE probes according to the following equation:

(1) $$\begin{array}{c} {\text{QY}}_{\text{sample}}={\text{QY}}_{\text{ref}}\times \frac{{\text{slope}}_{\text{sample}}}{{\text{slope}}_{\text{ref}}}\times {\left(\frac{n_{\text{sample}}}{n_{\text{ref}}}\right)}^{2}, \end{array}$$

where QY_sample_ is the QY of AIE probes, QY_ref_ is the QY of IR-26 (0.5%), and *n*_sample_ and *n*_ref_ are the refractive indices of AIE probes and IR-26.

### 4.5. *In Vitro* Cellular Studies

Human umbilical vein endothelial cells were cultured at 37°C in Dulbecco's modified Eagle medium (DMEM) containing 10% fetal bovine serum and 1% penicillin/streptomycin with a humidified environment containing 5% CO_2_. Human umbilical vein endothelial cells (1.0 × 10^4^ cells per well) were seeded in 96-well plates and incubated with various concentrations of AIE probes (0 *μ*g mL^−1^, 3.125 *μ*g mL^−1^, 6.25 *μg* mL^−1^, 12.5 *μ*g mL^−1^, 25 *μ*g mL^−1^, 50 *μ*g mL^−1^, 100 *μ*g mL^−1^, and 200 *μ*g mL^−1^) for 24 h. The whole blood of monkey was collected, centrifuged (1500 rpm, 3 min) to separate the red blood cells, and washed (1500 rpm, 3 min) with PBS three times. 10% RBC (*v*/*v*, in PBS) was incubated with AIE probes (0 *μ*g mL^−1^, 25 *μ*g mL^−1^, 50 *μ*g mL^−1^, 100 *μ*g mL^−1^, 150 *μ*g mL^−1^, 200 *μ*g mL^−1^, 300 *μ*g mL^−1^, and 400 *μ*g mL^−1^) at 37°C for 3 h, respectively. After centrifugation, the supernatant of the suspensions was collected and analyzed by a UV-vis spectrometer at 541 nm.

### 4.6. Animal Studies

Four cynomolgus monkeys (male, 3–4 kg, 4–5 years) were obtained from the Huazhen Laboratory Animal Breeding Centre (Conghua, Guangzhou, China). All animal experiment procedures were performed in compliance with the Institutional Animal Care and Use Committee of Huazhen Laboratory Animal Breeding Centre, China (no. HZ-AEC-TORM-051). The cynomolgus monkeys were housed individually in stainless steel cages (20–22°C, 40–60% relative humidity, 12-hour light-dark cycle, 15 air changes per hour) and fed a commercial monkey diet. Fresh fruits were also supplemented, and sufficient water was available. Research staff inspected the monkeys three times each day. Cynomolgus monkeys remained healthy and active, and food intake and body weight were normal. After collecting fasting blood samples, AIE probes (1 mg mL^−1^) dispersed in 0.9% NaCl were filter-sterilized before injection. Blood samples for the analysis of clinical chemistry and clinical hematology analyses were collected on days 0, 1, 2, 4, and 7 of each injection cycle. Body mass, temperature, appearance, and exploratory behavior were recorded at the same time. The clinical chemistry and clinical hematology analyses were carried out on the pocH-100ivD fully automated hematology analyzer (Sysmex Corporation, Kobe, Japan).

### 4.7. Histological Analysis

A randomly selected cynomolgus monkey was euthanized at the end of 36 days postinjection of AIE probes. The heart, liver, spleen, lungs, kidneys, colon, muscle, lymph nodes, and brain were collected and fixed with 4% paraformaldehyde. The tissue slices were stained with hematoxylin and eosin and observed on the digital (Pannoramic Desk; 3D Histech, Budapest, Hungary). Tissue sections were examined by two independent clinical pathologists.

### 4.8. *In Vivo* NIR-II Fluorescence Imaging

NIR-II fluorescence imaging was performed using NIR-OPTICS Series III 900/1700 small animal imaging system. The fluorescence imaging parameters were 808 nm laser with a power density of 30 mW cm^−2^, 1250 nm long-pass filter, and 200 ms exposure time using a 640 × 512 pixel two-dimensional InGaAs/SWIR camera (Photonic Science, UK), with an intravenous dose of 2 mg kg^−1^.

## Supplementary Materials

Supplementary 1 Supplementary 1. Fig. S1: synthetic route of the TTB molecular. Fig. S2: 1H NMR spectrum of 2 in CDCl3. Fig. S3: 13C NMR spectrum of 2 in CDCl3. Fig. S4: high-resolution mass spectrum of 2. Fig. S5: 1H NMR spectrum of TTB in CDCl3. Fig. S6: 13C NMR spectrum of TTB in CDCl3. Fig. S7: high-resolution mass spectrum of TTB. Fig. S8: the particle size of AIE probes over storage time. Fig. S9: measurement of the quantum yield of AIE probes using IR26 as reference. Fig. S10: photostability of AIE probes in PBS under the continuous natural light irradiation for 30 days. Fig. S11: body weight of cynomolgus monkeys during the experiment period. Fig. S12: body temperature of cynomolgus monkeys during the experiment period. Fig. S13: fluorescence signals of feces collected at different time points. Fig. S14: ex vivo NIR-II fluorescence imaging of the liver, spleen, and lymph nodes of AIE probe-treated cynomolgus monkey at the end of a 35-day postinjection. Fig. S15: ex vivo NIR-II fluorescence imaging of the muscle, stomach, brain, heart, intestine, kidney, and lung of AIE probe-treated cynomolgus monkey at the end of a 35-day postinjection. Fig. S16: dynamic NIR-II fluorescence imaging of blood flow at with 5 frames per second. Fig. S17: dynamic NIR-II fluorescence imaging of blood flow at with 5 frames per second. Table S1: summary of reported NIR-II AIE probes for in vivo fluorescence imaging.

Supplementary 2 Video 1: NIR-II vascular perfusion imaging.

Supplementary 3 Video 2: NIR-II vascular imaging at 1.5-centimeter depth.
