# Supplementary material for: Centimeter-Deep NIR-II Fluorescence Imaging with Nontoxic AIE Probes in Nonhuman Primates
Source: Research (Wash D C). 2020 Sep 26;2020:4074593. doi: 10.34133/2020/4074593 (PMC7533907; doi:10.34133/2020/4074593)
Supplement: Supplementary 1 — Fig. S1: synthetic route of the TTB molecular. Fig. S2: 1H NMR spectrum of 2 in CDCl3. Fig. S3: 13C NMR spectrum of 2 in CDCl3. Fig. S4: high-resolution mass spectrum of 2. Fig. S5: 1H NMR spectrum of TTB in CDCl3. Fig. S6: 13C NMR spectrum of TTB in CDCl3. Fig. S7: high-resolution mass spectrum of TTB. Fig. S8: the particle size of AIE probes over storage time. Fig. S9: measurement of the quantum yield of AIE probes using IR26 as reference. Fig. S10: photostability of AIE probes in PBS under the continuous natural light irradiation for 30 days. Fig. S11: body weight of cynomolgus monkeys during the experiment period. Fig. S12: body temperature of cynomolgus monkeys during the experiment period. Fig. S13: fluorescence signals of feces collected at different time points. Fig. S14: ex vivo NIR-II fluorescence imaging of the liver, spleen, and lymph nodes of AIE probe-treated cynomolgus monkey at the end of a 35-day postinjection. Fig. S15: ex vivo NIR-II fluorescence imaging of the muscle, stomach, brain, heart, intestine, kidney, and lung of AIE probe-treated cynomolgus monkey at the end of a 35-day postinjection. Fig. S16: dynamic NIR-II fluorescence imaging of blood flow at with 5 frames per second. Fig. S17: dynamic NIR-II fluorescence imaging of blood flow at with 5 frames per second. Table S1: summary of reported NIR-II AIE probes for in vivo fluorescence imaging. [file 4074593.f1.docx]

**Centimeter-Deep NIR-II Fluorescence Imaging with Nontoxic AIE Probes in Non-Human Primates**

Zonghai Sheng^1^, Yaxi Li^2^, Dehong Hu^1^, Tianliang Min^2^, Duyang Gao^1^, Jen-Shyang Ni^2^, Pengfei Zhang^1^, Yuenan Wang^4^, Xin Liu^1^, Kai Li^2^*, Hairong Zheng^1^* & Ben Zhong Tang^3^*

^1^ Paul C. Lauterbur Research Center for Biomedical Imaging, Shenzhen Key Laboratory of Ultrasound Imaging and Therapy, CAS key laboratory of health informatics, Institute of Biomedical and Health Engineering, Shenzhen Institute of Advanced Technology, Chinese Academy of Sciences, Shenzhen, 518055, P. R. China.

^2^ Department of Biomedical Engineering, Southern University of Science and Technology (SUSTech), Shenzhen, 518055, P. R. China.

^3^ Department of Chemistry, The Hong Kong University of Science & Technology, Clear Water Bay, Kowloon, Hong Kong, P. R. China.

^4^ Department of Radiation Oncology, National Cancer Center/National Clinical Research Center for Cancer/Cancer Hospital & Shenzhen Hospital, Chinese Academy of Medical Sciences and Peking Union Medical College, Shenzhen, 518055, P. R. China.

* Corresponding Authors: Ben Zhong Tang; tangbenz@ust.hk; Hairong Zheng; hr.zheng@siat.ac.cn; and Kai Li; lik@sustech.edu.cn

**Fig. S1.** Synthetic route of TTB.


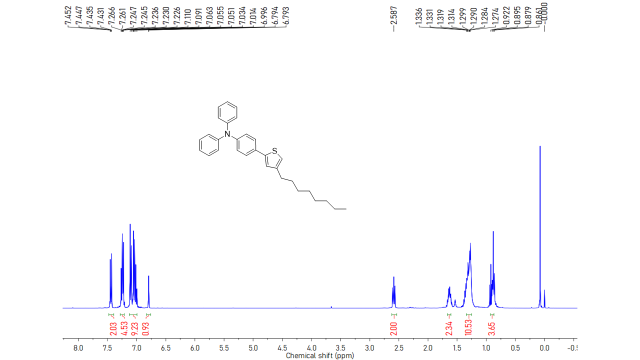


**Fig. S2** ^1^H NMR spectrum of **2** in CDCl_3_.


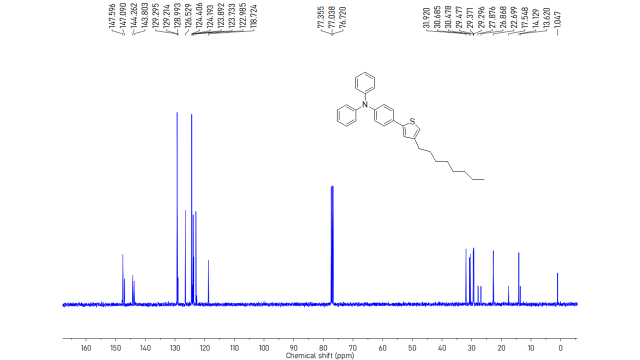


**Fig. S3** ^13^C NMR spectrum of **2** in CDCl_3_.


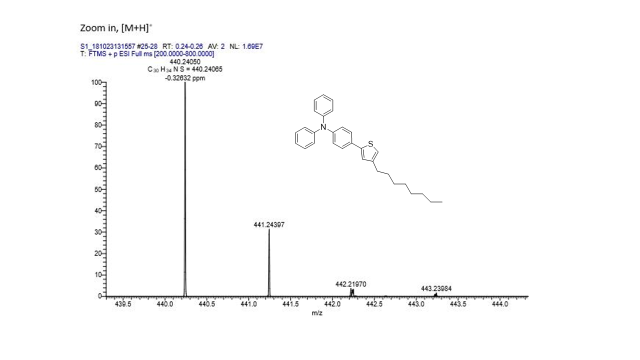


**Fig. S4** High-resolution mass spectrum of **2**.


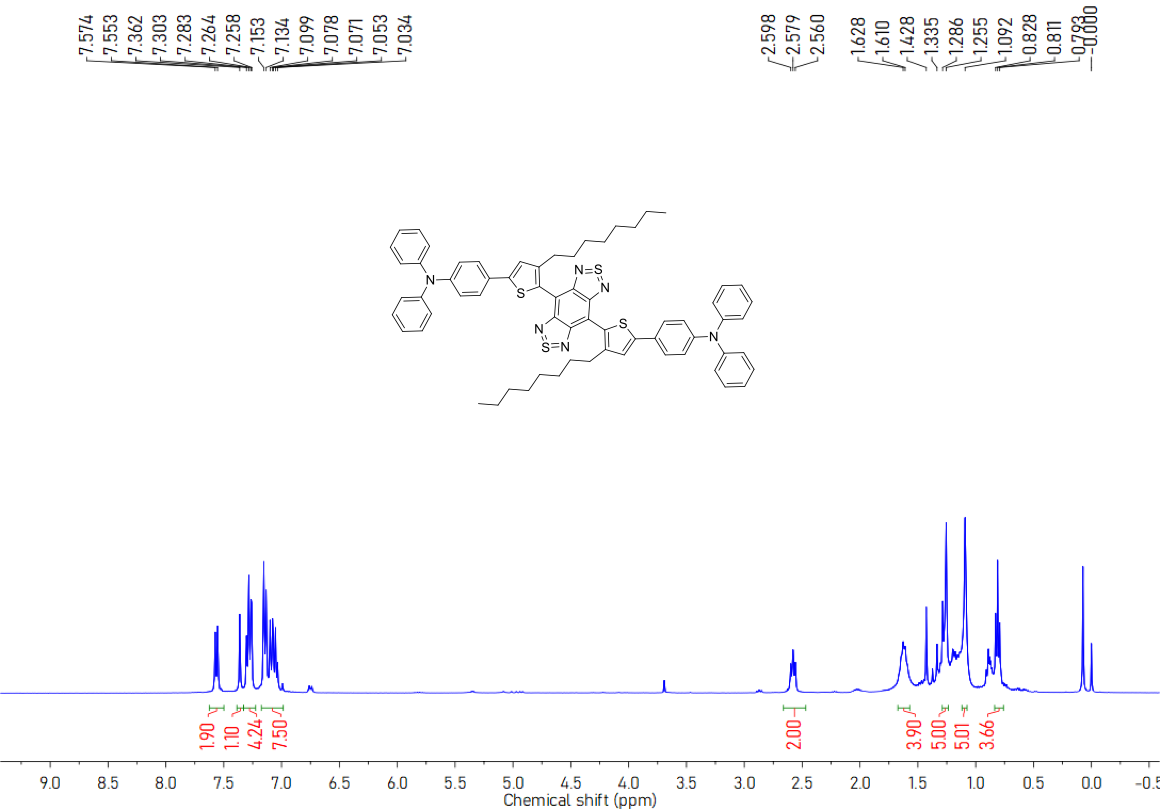


**Fig. S5** ^1^H NMR spectrum of TTB in CDCl_3_.


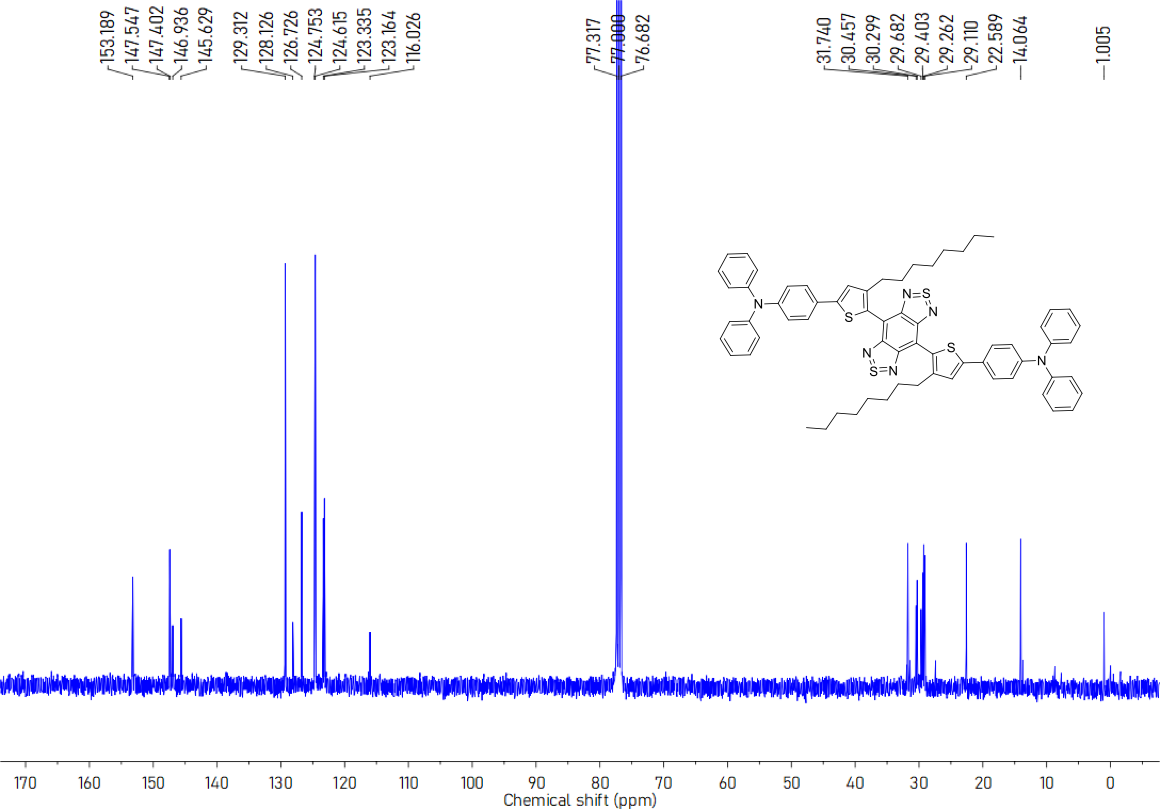


**Fig. S6** ^13^C NMR spectrum of TTB in CDCl_3_.


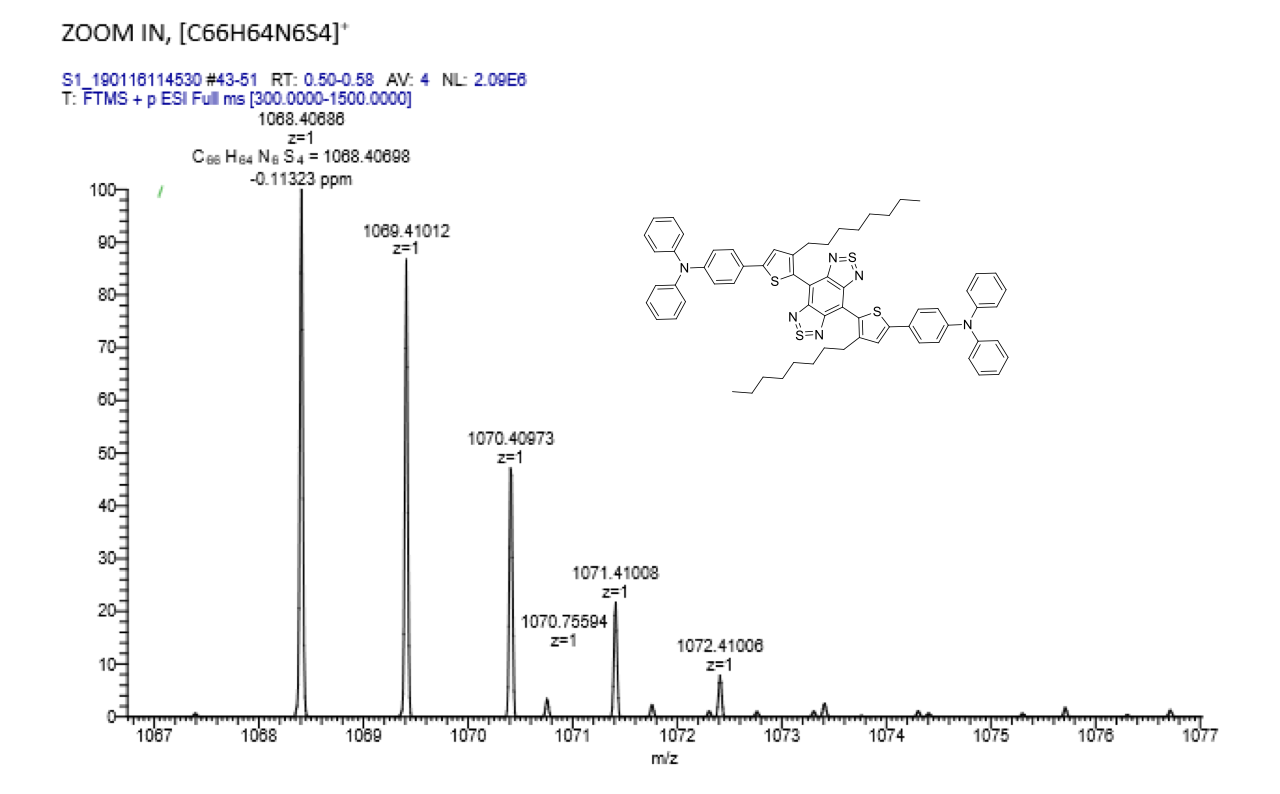


**Fig. S7** High-resolution mass spectrum of TTB.


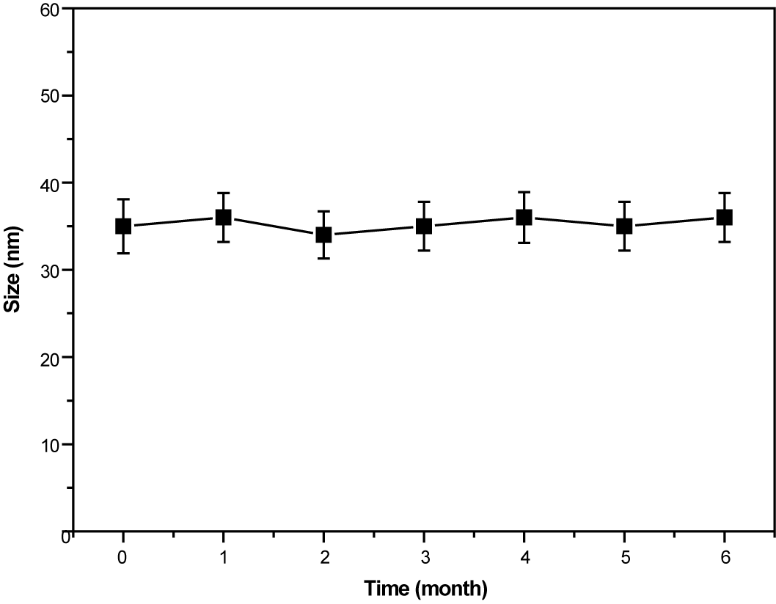


**Fig. S8** The particle size of AIE probes over storage time.


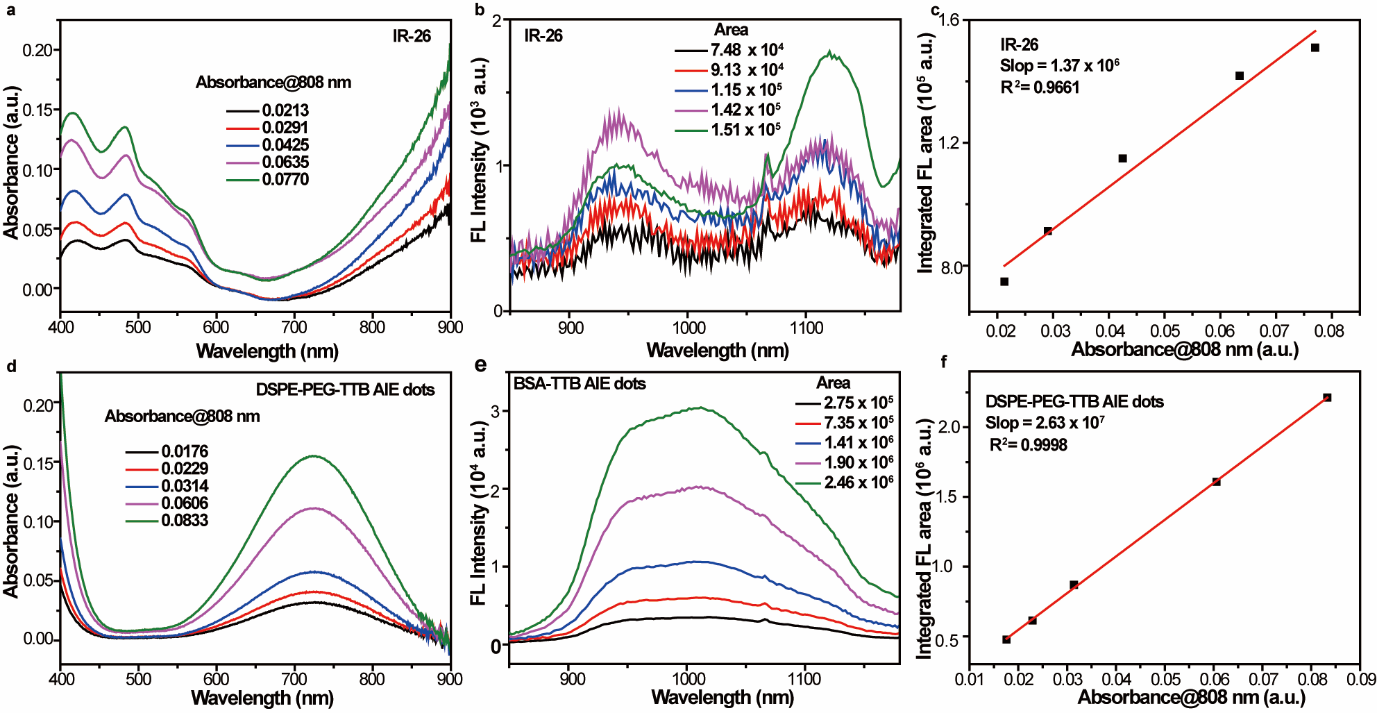


**Fig. S9** Measurement of the quantum yield of AIE probes using IR26 as reference. (a) UV-Vis-NIR absorption spectra of IR-26 with different concentrations dissolved in 1,2-dichloroethane (DCE). (b) NIR emission spectra of the IR-26 solutions under 808 nm laser irradiation. (c) The fitted linear function between the absorption values and emission area of the IR-26. (d) UV-Vis-NIR absorption spectra of AIE probes with different concentrations dissolved in PBS. (e) NIR-II emission spectra of the AIE probes solutions under 808 nm laser irradiation. (f) The fitted linear relationship between the absorption values and emission area of the AIE probes.


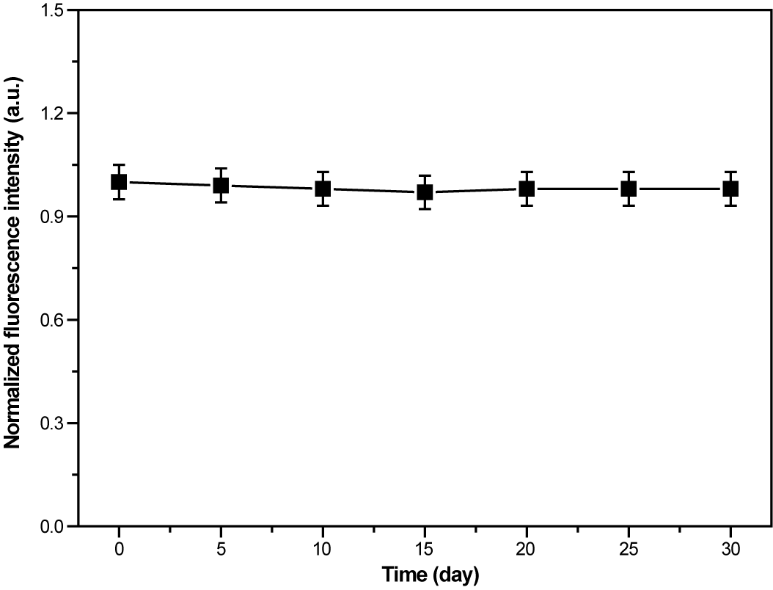


**Fig. S10** Photostability of AIE probes in PBS under the continuous natural light irradiation for 30 days.


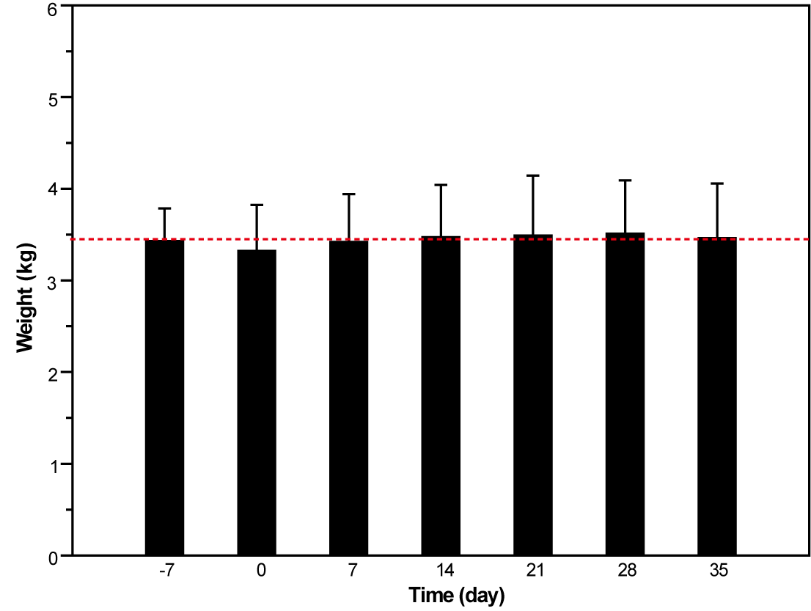


**Fig. S11** Body weight of cynomolgus monkeys during the experiment period. The red dashed line indicates the average value of the control group (n=3).


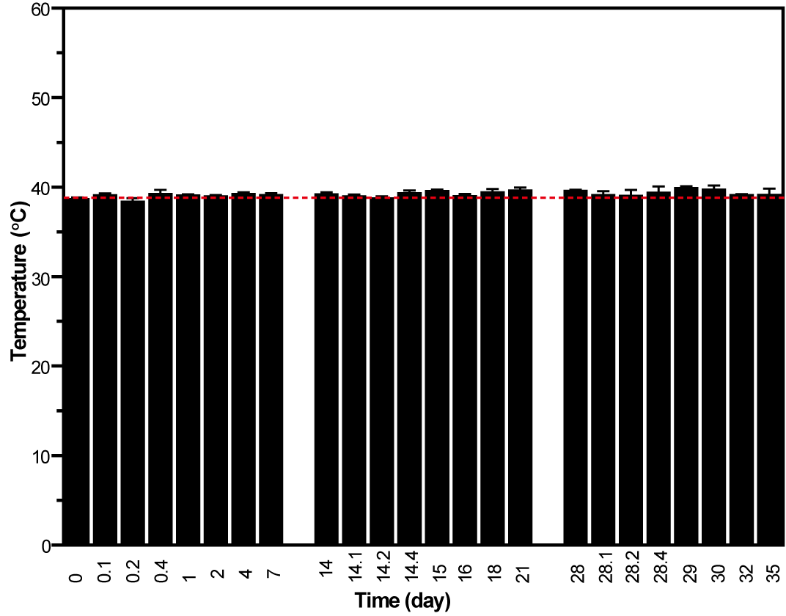


**Fig. S12** Body temperature of cynomolgus monkeys during the experiment period. The red dashed line indicates the average value of the control group (n=3).


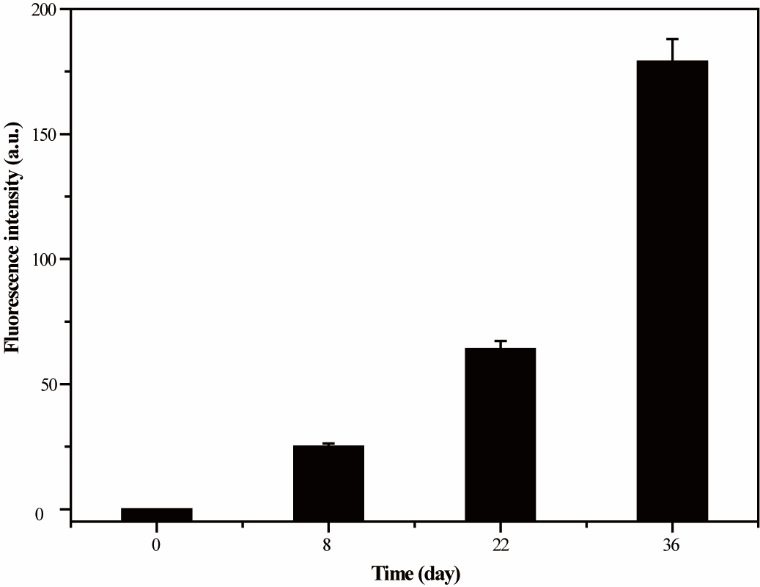


**Fig. S13** Fluorescence signals of feces collected at different time points.


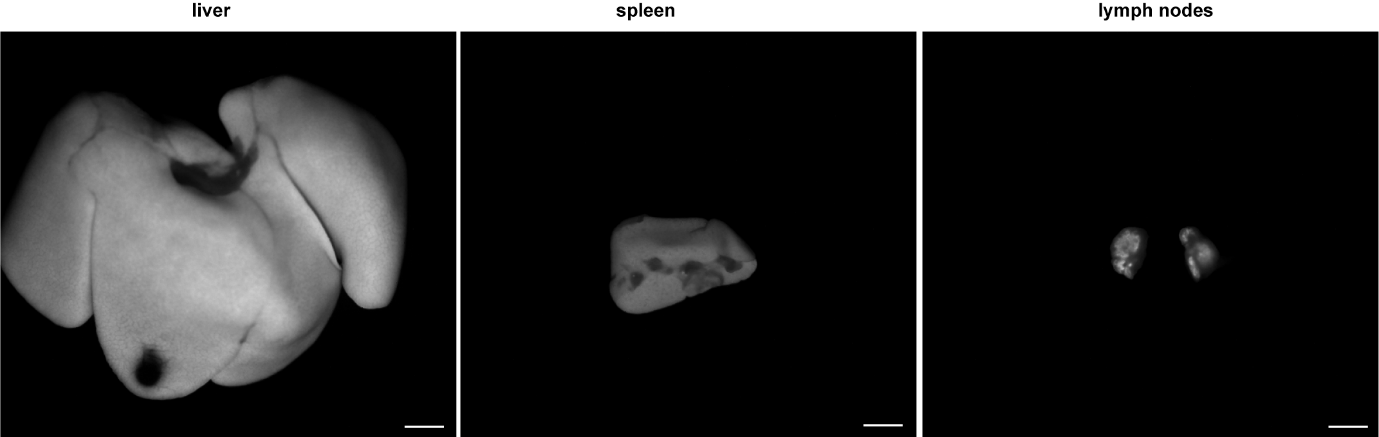


**Fig. S14** *Ex vivo* NIR-II fluorescence imaging of liver, spleen, and lymph nodes of AIE probes treated cynomolgus monkey at the end of 35 days post-injection. Scale bar: 1.0 cm.


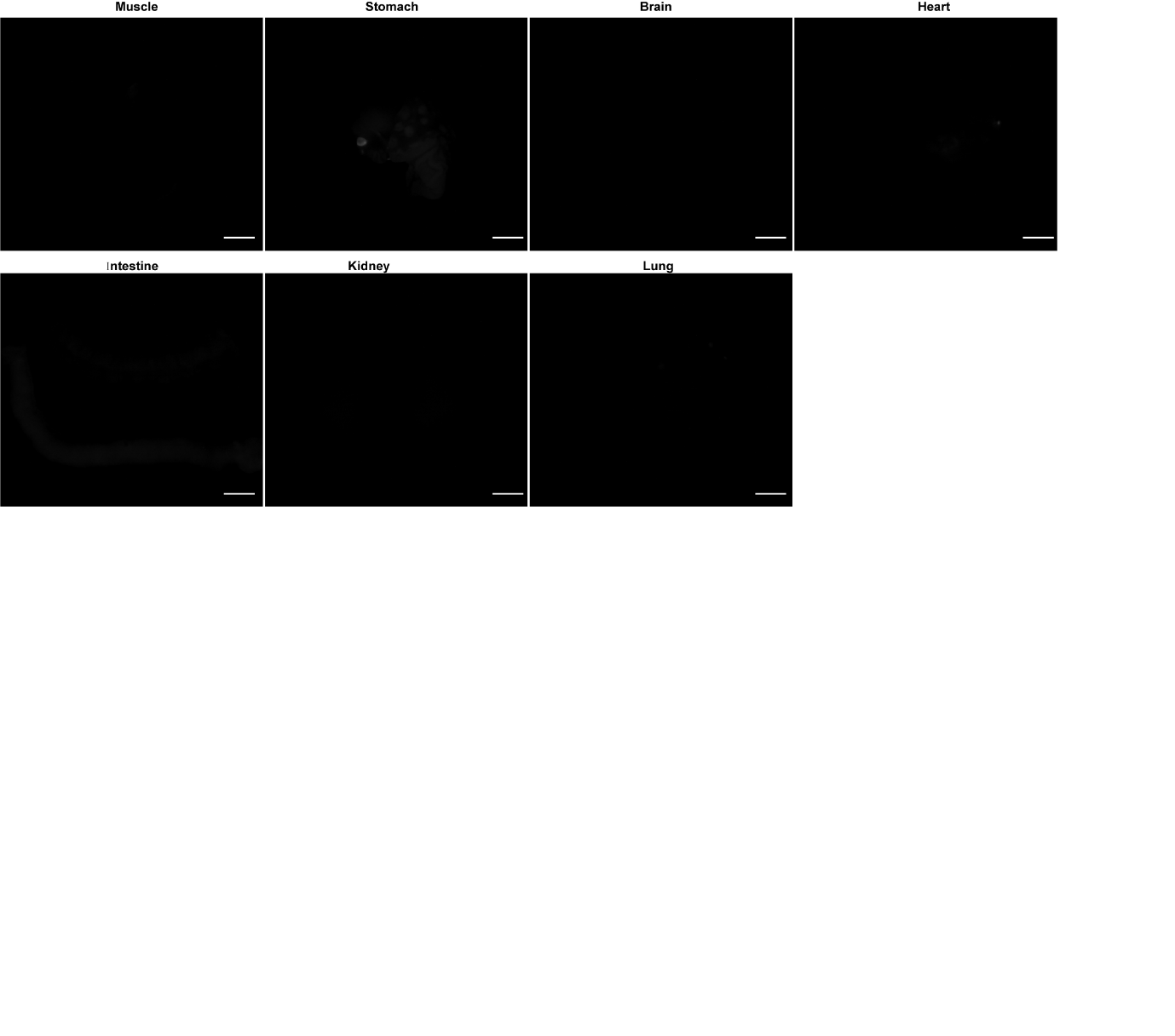


**Fig. S15** *Ex vivo* NIR-II fluorescence imaging of muscle, stomach, brain, heart, intestine, kidney, and lung of AIE probes treated cynomolgus monkey at the end of 35 days post-injection. No NIR-II fluorescence was detected. Scale bar: 1.0 cm.


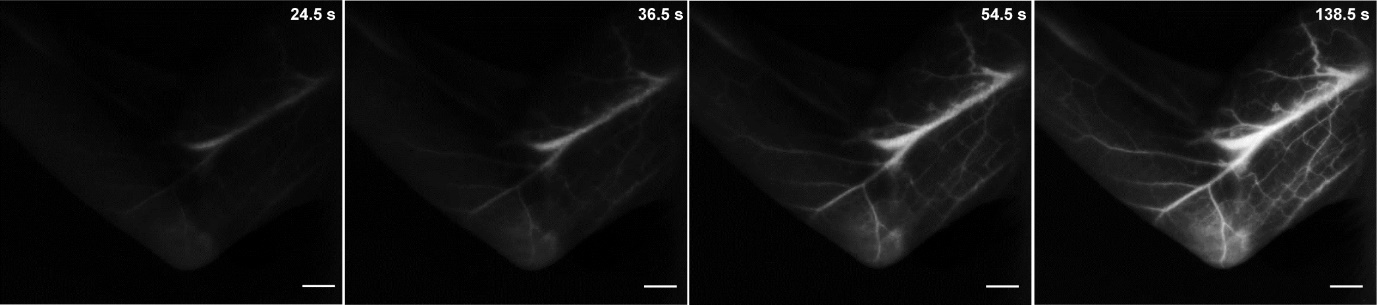


**Fig. S16** Dynamic NIR-II fluorescence imaging of blood flow at with 5 frames per second. Dose: 2 mg Kg^-1^, Exposure time: 200 ms, the power of 808-nm laser excitation (30 mW cm^-2^), Scale bar: 0.5 cm.


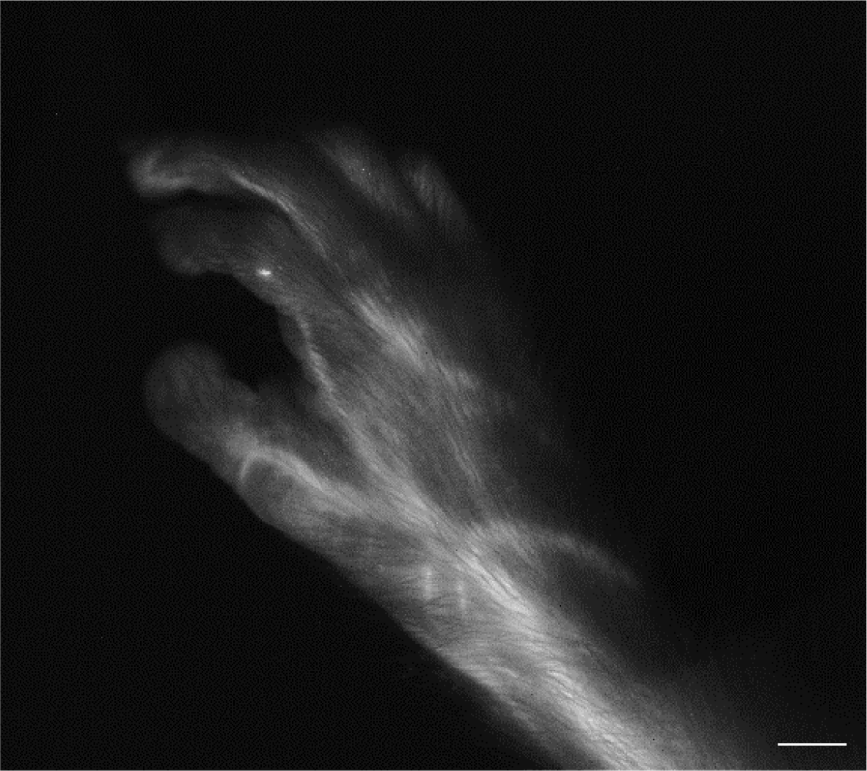


**Fig. S17** Dynamic NIR-II fluorescence imaging of blood flow at with 5 frames per second. NIR-II fluorescence imaging of vascular perfusion of normal cynomolgus without depilation. Dose: 2 mg Kg^-1^, Exposure time: 200 ms, the power of 808-nm laser excitation (30 mW cm^-2^), Scale bar: 0.5 cm.

**Table S1.** Summary of reported NIR-II AIE probes for *in vivo* fluorescence imaging.

| **Year** | **Molecular Structure** | **Absorption Maximum (nm)** | **Emission Maximum (nm)** | **QY (%)** | **Ref** |
| --- | --- | --- | --- | --- | --- |
| 2018 | 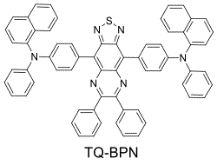 | 630 | 808 | 2.8 | [1] |
| 2018 | 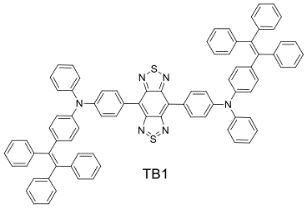 | 740 | 975 | 6.2 | [2] |
| 2018 | 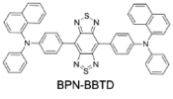 | 700 | 949 | 1.8 | [3] |
| 2019 | 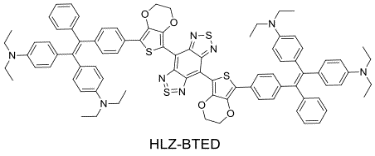 | 805 | 1034 | 0.18 | [4] |
| 2019 | 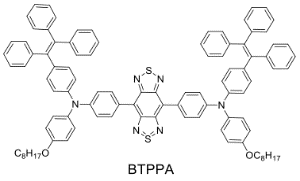 | 761 | 1013 | 9.9 | [5] |
| 2019 | 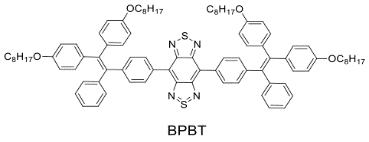 | 715 | 897 | 5.8 | [6] |
| 2019 | 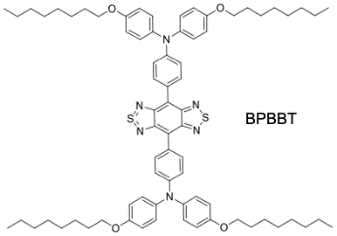 | - | 1050 | 1.45 | [7] |
| 2020 | 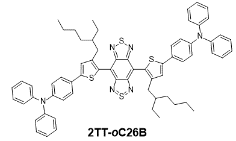 | 730 | 1031 | 11.5 | [8] |
| 2020 | 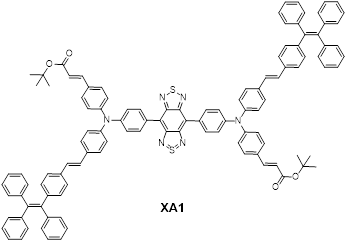 | 400，780 | 1000 | 14.5% | [9] |
| 2020 | 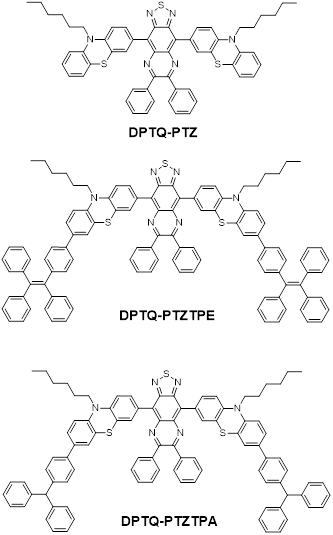 | 639, 661, 642 | 928, 930, 897 | 0.16%, 0.22%, 0.29% | [10] |

1. Qi J, Sun C, Zebibula A, Zhang H, Kwok RTK, Zhao X*, et al.* Real-time and high-resolution bioimaging with bright aggregation-induced emission dots in short-wave infrared region. *Adv. Mater.* **30**, 1706856 (2018).

2. Sheng Z, Guo B, Hu D, Xu S, Wu W, Liew WH*, et al.* Bright aggregation-induced-emission dots for targeted synergetic NIR-II fluorescence and NIR-I photoacoustic imaging of orthotopic brain tumors. *Adv. Mater.* **30**, 1800766 (2018).

3. Alifu N, Zebibula A, Qi J, Zhang H, Sun C, Yu X*, et al.* Single-molecular Near-Infrared-II theranostic systems: ultrastable aggregation-induced emission nanoparticles for long-term tracing and efficient photothermal therapy. *ACS Nano* **12**, 11282-11293 (2018).

4. Lin J, Zeng X, Xiao Y, Tang L, Nong J, Liu Y*, et al.* Novel near-infrared II aggregation-induced emission dots for in vivo bioimaging. *Chem. Sci.* **10**, 1219-1226 (2019).

5. Wu W, Yang Y-Q, Yang Y, Yang Y-M, Wang H, Zhang K-Y*, et al.* An organic NIR-II nanofluorophore with aggregation-induced emission characteristics for in vivo fluorescence imaging. *Int. J. Nanomed.* **14**, 3571-3582 (2019).

6. Wu W, Yang Y, Yang Y, Yang Y, Zhang K, Guo L*, et al.* Molecular engineering of an organic NIR-II fluorophore with aggregation-induced emission characteristics for in vivo imaging. *Small* **15**, 1805549 (2019).

7. Gao S, Wei G, Zhang S, Zheng B, Xu J, Chen G*, et al.* Albumin tailoring fluorescence and photothermal conversion effect of near-infrared-II fluorophore with aggregation-induced emission characteristics. *Nat. Commun.* **10**, 1-15 (2019).

8. Li Y, Cai Z, Liu S, Zhang H, Wong ST, Lam JW*, et al.* Design of AIEgens for near-infrared IIb imaging through structural modulation at molecular and morphological levels. *Nat. Commun.* **11**, 1-10 (2020).

9. Xu P, Kang F, Yang W, Zhang M, Dang R, Jiang P*, et al.* Molecular engineering of a high quantum yield NIR-II molecular fluorophore with aggregation-induced emission (AIE) characteristics for in vivo imaging. *Nanoscale* **12**, 5084-5090 (2020).

10. Li S, Yin C, Wang R, Fan Q, Wu W, Jiang X. Second Near-Infrared aggregation-induced emission fluorophores with phenothiazine derivatives as the donor and 6,7-diphenyl-[1,2,5]thiadiazolo[3,4-g]quinoxaline as the acceptor for in vivo imaging. *ACS. Appl. Mater. Inter.* **12**, 20281-20286 (2020).
